# The need for better AD animal models

**DOI:** 10.3389/fphar.2014.00227

**Published:** 2014-10-24

**Authors:** Miguel Medina, Jesús Avila

**Affiliations:** ^1^Centro de Investigación Biomédica en Red de Enfermedades Neurodegenerativas (CIBERNED)Madrid, Spain; ^2^Centro de Biología Molecular “Severo Ochoa” CSIC-UAMMadrid, Spain

**Keywords:** Alzheimer, amyloid, dementia, drug development, transgenics, neurodegeneration, tau, therapy

Drug development for Alzheimer's disease (AD) has proven to be particularly difficult. Only five symptomatic drugs have been approved for its treatment between 1993 and 2003. Despite great efforts on research and huge investments on drug development, no new treatments have been approved for AD since 2003. In particular, drug development success rate has been staggering low with many small molecules and immunotherapies failing to show efficacy or superiority over placebo in late clinical trials (Cummings et al., 2014). Franco and Cedazo-Minguez (2014) have recently addressed this critical issue by underlying some highly relevant open questions, some of which are briefly discussed herein, especially regarding the lack of predictive capability shown so far by the available animal models for the discovery of novel therapies.

Age-related neurodegenerative disorders, including AD are largely human-specific pathologies. Even though some human brain aging aspects can be similarly observed in aged non-human primates and perhaps also in other mammalian species, they do not readily develop the full set of neuropathological and/or clinical phenotypic features observed in the human pathology. Nevertheless, cell and animal models, including genetically engineered non-mammalian species (*C. elegans*, *D. melanogaster*, zebra fish) have been remarkably useful in dissecting the basic disease mechanisms and for the screening of compounds directed toward specific molecular pathways involved in AD.

Historically, the AD field has been long dominated by the characteristic pathological hallmarks described by Alois Alzheimer over a century ago: intraneuronal neurofibrillary tangles (made up of tau filaments) and extracellular senile plaques (made up of Aβ peptide aggregates). A key question remains to be answered though: how these autopsy findings (endpoints of the process) mechanistically relate with the origin or cause of the disease? So far, therapeutic strategies have been mainly driven by pathology, with most drug development efforts in the last twenty years having been directed toward Aβ, essentially focused on the amyloid cascade hypothesis, with disappointing results until now. On the other hand, tau-based strategies (and targets other than Aβ) have received little consideration until very recently even though extensive tau pathology is crucial to the disease and that recent genetic studies have discovered mutations within the tau gene leading to frontotemporal dementia, demonstrating that tau dysfunction *per se*, in the absence of amyloid pathology, is enough to cause neurodegeneration and clinical dementia.

During the last decade, the study of the genetics of familial AD (FAD) has provided a wealth of knowledge on the elements that affect the molecular mechanisms underlying AD pathogenesis and established that, apart from the age of onset, sporadic AD is clinically and neuropathologically similar to the most common familial forms. These phenotypic resemblance has inspired the development of a wide variety of genetically modified cellular and animal models, based on the mutations present in FAD. Genetics of FAD has also provided the strongest support for a critical role for Aβ in AD pathophysiology, but the fact that some of the mutations in presenilin 1 (PS1), the most commonly mutated gene in FAD, are not directly related to Aβ pathology suggests that there is something else to it.

Albeit they might not be the best species for mimicking the human disease, transgenic mice are still undoubtedly the most popular and extensively used animal models for studying AD. The studies carried on in animal models have also rendered invaluable information on the pathogenesis and pathophysiology of AD including, for instance, novel insights into the molecular mechanisms underlying the pathological aggregation of pivotal proteins, the pathways toward neuronal damage, the contribution of genetic risk factors and the role of neuroinflammation in neurodegeneration. Yet, these models appears to have contributed only partially to shed light on the actual mechanisms triggering the disease, thus preventing a true translation into new therapies, diagnosis and prevention. Even though an impressive amount of knowledge has been generated from the use of animal models, it has only marginally enriched our therapeutic potential. Actually, the hopes often raised by encouraging preclinical results have evanesced when the new strategies have been tested in clinical trials.

Besides an excessive focus on the amyloid cascade hypothesis as paramount in AD pathogenesis and a lack of integration of a large body of other data relevant to the emergence of clinical AD, methodological issues related to clinical trials might all have contributed for the failure to translate successful results from animal models into clinical trials. The latter has been recently started to be addressed by the new NIA-AA guidelines for diagnostic of AD (McKhann et al., 2011) and mild cognitive impairment (MCI) (Albert et al., 2011) as well as the recognition of preclinical AD as a newly defined stage (Sperling et al., 2011) based on recent evidence showing some biomarker changes many years prior to the onset of clinical symptoms. All this has led to an increasing interest in testing drugs (that already failed in late clinical trials) in prevention trials in FAD subjects, with the hope that beginning the treatment earlier might prove efficacious (Reiman et al., 2011; Carrillo et al., 2013). Furthermore, development of solid, reproducible biomarkers may help the current clinical outcomes based solely on cognition and facilitate drug development of disease-modifying drugs. On the other hand, as stated by Franco and Cedazo-Minguez (2014) in Table 2, limited knowledge of the specific features of the countless animal models generated may also have contributed to the lack of success in translating preclinical research into clinical application.

A number of complexity elements must be kept in mind when trying to model AD in animals: (i) Genetics (mutations, risk-associated common variants sporadic forms, epigenetics); (ii) Environment (toxins, diet, stress, social interactions, infections); and (iii) Aging (metabolic changes, hormones, genomic instability, accumulation of damaging insults). Most of the currently used models are not able to recreate the complexity of the human disease since they only take into account these factors individually. However, the combined analysis of these factors and the evaluation of their exact contribution to the development of the pathology is most suitable in animal models and cannot be fully accomplished in cellular or *ex-vivo* models. Thus, in order to better figure out the significance of the interplay among different neural cells and circuits, the next generation of animal models must reproduce the human disease more precisely. Together with further understanding of the molecular mechanisms involved and better, clinically relevant readouts it should greatly help improving predictability in translating efficacy results in animal models to the clinical setting.

Finally, in light of recent developments of *in silico* approaches to modeling of at least some aspects of brain function, we would like to further emphasize the essential role of experimental models to dissect pathogenic mechanisms and to support preclinical drug development.

## Conflict of interest statement

The authors declare that the research was conducted in the absence of any commercial or financial relationships that could be construed as a potential conflict of interest.
